# Essential structural and experimental descriptors for bulk and grain boundary conductivities of Li solid electrolytes

**DOI:** 10.1080/14686996.2020.1824985

**Published:** 2020-10-19

**Authors:** Yen-Ju Wu, Takehiro Tanaka, Tomoyuki Komori, Mikiya Fujii, Hiroshi Mizuno, Satoshi Itoh, Tadanobu Takada, Erina Fujita, Yibin Xu

**Affiliations:** a Center for Materials Research by Information Integration (CMI2), Research and Services Division of Materials Data and Integrated System (Madis), National Institute for Materials Science (NIMS), Tsukuba, Japan; b International Center for Young Scientists (ICYS), National Institute for Materials Science (NIMS), Tsukuba, Japan; c Technology Division, Innovation Promotion Sector, Panasonic Corporation, Osaka, Japan

**Keywords:** Ionic conductivity, machine learning, grain boundary, ionic conductor, Li battery, grain size, descriptor, 107 Glass and ceramic materials, 404 Materials informatics / Genomics, 206 Energy conversion / transport / storage / recovery

## Abstract

We present a computational approach for identifying the important descriptors of the ionic conductivities of lithium solid electrolytes. Our approach discriminates the factors of both bulk and grain boundary conductivities, which have been rarely reported. The effects of the interrelated structural (e.g. grain size, phase), material (e.g. Li ratio), chemical (e.g. electronegativity, polarizability) and experimental (e.g. sintering temperature, synthesis method) properties on the bulk and grain boundary conductivities are investigated via machine learning. The data are trained using the bulk and grain boundary conductivities of Li solid conductors at room temperature. The important descriptors are elucidated by their feature importance and predictive performances, as determined by a nonlinear XGBoost algorithm: (i) the experimental descriptors of sintering conditions are significant for both bulk and grain boundary, (ii) the material descriptors of Li site occupancy and Li ratio are the prior descriptors for bulk, (iii) the density and unit cell volume are the prior structural descriptors while the polarizability and electronegativity are the prior chemical descriptors for grain boundary, (iv) the grain size provides physical insights such as the thermodynamic condition and should be considered for determining grain boundary conductance in solid polycrystalline ionic conductors.

## GRAPHICAL ABSTRACT

**Figure:**
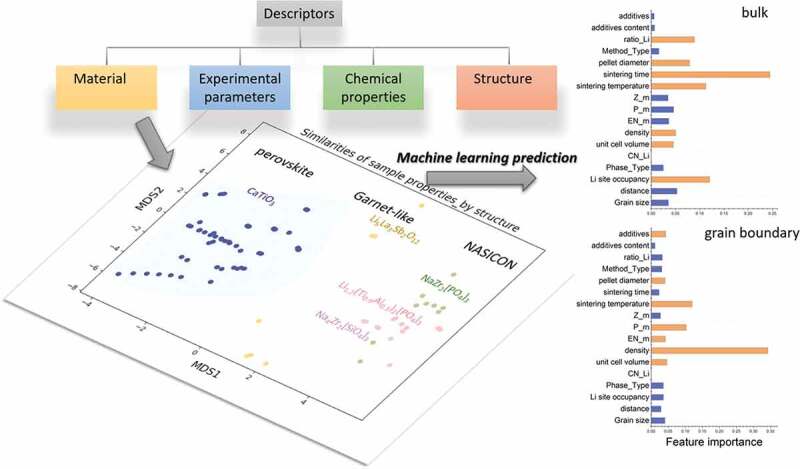

## 1. Introduction

Rechargeable Li batteries are widely applied in portable devices, and their applications have extended to energy storage and electric vehicle industries. Li solid electrolytes might replace organic electrolytes, which are flammable and unstable to leakage, with electrodes that meet the high safety standards of electric vehicles. This development would realize all-solid-state Li batteries. Besides improving the safety and sustainability of higher cycles, Li solid electrolytes with a lithium-metal anode enable a wider electrochemical window than conventional electrolytes [1]. The electrochemical window is an important characteristic of the potential range of an electrolyte, obtained by subtracting the reduction potential of the cathode from the oxidation potential of the anode. A wide electrochemical window is beneficial in applications with high energy density, such as electric vehicles and electrical energy-storage systems.

The ionic transport efficiency of a Li solid electrolyte is determined by its total ionic conductivity, which is composed of the bulk (i.e. grain interior) and grain boundary conductivities. Typically, the ionic conductivities are measured by impedance spectroscopy over a wide frequency range to resolve their various contributions, whereas the bulk and grain boundary conductivities are verified by adequate equivalent-circuit models [2,3]. Lilley et al. and Breuer et al. reported that at temperatures well below room temperature (~173 K) the bulk conductivity contributions are easily separated from the grain boundary response [2,4]. As the bulk conductivities are usually more than one order of magnitude higher than the grain boundary conductivities [5–12], the grain boundary conductivities are the limiting factor of ionic transport. However, the dominant factors of the individual bulk and grain boundary conductivities have not been comprehensively elucidated.

In previous studies, the factors affecting ionic conductivities were divided into three closely related groups: (1) the microstructure, which varies dominantly with the experimental conditions, (2) additives such as Li_3_PO_4_ [13], Al_2_O_3_ [14] and excess Li [15], which change the microstructure and may induce the blocking effect, and (3) chemical properties, which depend on the atomic compositions. The most widely discussed factor, microstructure, is proposed to increase the ionic conductivity by reducing the number of defects and low-conductive boundary region or by inducing phase transitions. An example is the tetragonal–cubic transition in sintered Li_7_La_3_Zr_2_O_12_ [16,17]. Microstructures such as the secondary phase, grain size distribution and short-circuited grain boundaries [2] are thought to cause inhomogeneous conduction paths of the grain boundaries across polycrystalline samples, which explicitly depend on the experimental conditions such as the sintering temperature [3]. Meanwhile, additives affect the sintered density and suppress the secondary phases by tuning the additive content, thus changing the ionic conductivities [18]. Also, sintering often cause ion segregation into the grain boundaries [19]. For example, adding excess Li improves the structural contact among the grains by segregating ions at the boundaries, thus reducing the activation energy of the grain boundary conductivity and increasing the concentration of conductive Li ions. The end result is improved ionic conduction [15]. Other reports have indicated that ion segregation at the grain boundaries creates an electrostatic barrier (a space-charge layer) that blocks ionic transport, with adverse effects on the bulk and grain boundary conductivities [5,20]. Accordingly, the experimental conditions affect the microstructures, whereas the additives affect the microstructure and the formation of barriers such as space-charge layers. Finally, the chemical properties (such as electronegativity and polarizability) provide information on the interaction between the Li ions and their neighboring ions. Polarizable anion frameworks or anisotropic polarizability of the anions enhance the migration of Li ions [21,22]. The chemical properties further depend on the material, structural and experimental properties mentioned above [23,24].

**Figure 1.**
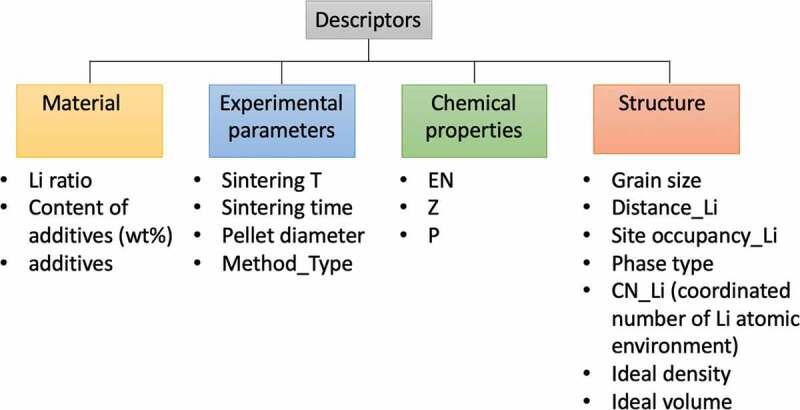
Illustration of descriptor categories. The 17 descriptors are grouped by material, experimental, chemical and structural properties collected from published papers [8,10–12,14,15,30–42] and AtomWork-Adv by NIMS (https://atomwork-adv.nims.go.jp/) [45]

The exploration and analysis of new solid Li conductors have relied on numerous trial-and-error experimental searches over the past decades. More recently, the prediction and exploration of fast solid ionic conductors have been assisted by machine learning and computational screening. The main descriptors in computational methods are the structural features of single crystals, calculated from density functional theory [25–29]. However, as mentioned above, the structural properties are significantly related to other factors such as additives, chemical compositions and experimental conditions. Also, the individual descriptors of bulk and grain boundary conductivities are unclear. It is worth noting that all commonly used solid Li conductors are polycrystalline or glass-ceramic rather than single crystals; therefore, the key factors corresponding to the grains and their boundaries are essential knowledge for characterizing and developing Li solid electrolytes. The topology and geometry of the structures (i.e. the available sites for mobile Li ions, chemical characteristics, and the experimental conditions) are closely connected and inseparable, so deconvoluting their importance and correlation with ionic conductivities is not advised. Instead, we propose a computational approach for analyzing the effects of the material, structural, chemical, and experimental properties on the ionic conductivities of both bulk and grain boundaries. The approach categorizes the different properties and is carried out by machine learning. The descriptors and their categorizations in the machine learning model are detailed in the Methods section (Figure 1).

## 2. Methods

### 2.1. Descriptor

The descriptors were selected based on the (a) experimental, chemical and structural affecters of ionic conductivity (as mentioned in the Introduction), and (b) the balance between the data availability and descriptor importance. Regarding (b), fewer descriptors generate less explainable percentages and information from the model, but many descriptors limit the numbers of data for training. For instance, the grain size has low availability in physical datasets, and is not easily collected from reported scanning electron microscopy (SEM) or transmission electron microscopy (TEM) images, but is an important determiner of grain boundary conductance of single boundaries (Equation (11)). Descriptors with strong correlations among each other, which generally have detrimental effects on the prediction performance, were excluded. For instance, electronegativity and ionic potential are strongly correlated, so only the electronegativity was retained as a descriptor.

The final 17 descriptors applied in the machine learning were categorized into material, experimental, chemical and structural properties (see Figure 1). The additives (0: absent; 1: present), content of additives (wt%), sintering temperature (°C), sintering time (h), pellet diameter (mm), method type, grain size (μm) and phase type were collected along with the ionic conductivities from various published papers [8,10–12,14,15,30–42]. The method types were ball mill/solid-state (0), melt quench (1), liquid phase (2), and sol–gel techniques (3). The phase type was crystal (0) or glass ceramic (1). When not stated in the published works, the grain sizes were calculated from the averages of five grains in the SEM or TEM images.

The Li ratio was calculated as the ratio of the number of Li atoms to the total number of atoms of all elements in the system, e.g. the Li ratio of Li_0.35_La_0.55_TiO_3_ was 0.35/(0.35 + 0.55 + 1 + 3) = 0.0714. The chemical descriptors were calculated by multiplying the *i-*th elemental chemical property of the element *j* ($P_{i}^{\left(j\right)}$) by the atomic ratio of the element *j*, and summing up this product for all elements in the system except for Li and O. The result $D_{i}$, defining the pseudo-weighted average of the *i*-th atomic property, is given by Equation (1).

(1) $$D_{i}=\frac{1}{N^{\left(tot\right)}}\underset{j\neq Li,O}{\sum }N^{\left(j\right)}P_{i}^{\left(j\right)}$$

where the elemental index *j* runs over all elements in the system except for Li and O. Here, the *i*-th elemental property $P_{i}$ is defined as follows: $P_{1}$ = electronegativity (EN) [43], $P_{2}$ = polarizability (P) [44], and $P_{3}$ = atomic number (Z). $N^{\left(j\right)}$ is the number of the atoms of the element *j* and $N^{\left(tot\right)}=\underset{j}{\sum }N^{\left(j\right)}$ is the total number of the atoms of all elements in the system *including* Li and O. As an example, the electronegativity of Li_0.35_La_0.55_TiO_3_ is calculated by Equation (2):

(2) $$D_{1}=\frac{1}{0.35+0.55+1+3}\left\{0.55\times P_{1}^{\left(La\right)}+1\times P_{1}^{\left(Ti\right)}\right\}$$

We excluded Li and O from the summation in Equation (1) in order to strengthen the difference through other elements than Li and O because all the systems in the dataset include Li and O. Yet the difference through the composition of Li and O are still reflected in Equation (1) through $N^{\left(tot\right)}.$

The structural properties, namely, the average distance between Li and its neighboring ions (Distance_Li), the site occupancy of Li ions (Site occupancy_Li), the coordination number of Li in its atomic environment (CN_Li), the density, and the unit cell volume, were collected for each sample from AtomWork-Adv compiled by the National Institute for Materials Science (NIMS) (https://atomwork-adv.nims.go.jp/) [45]. The density (g/cm^3^) and unit cell volume (Å^3^) were the values per chemical formula unit, the ideal density and unit cell volume of a single crystal.

### 2.2. Machine learning model

As linear/nonlinear based regressors and considering the dataset size, we selected the least-absolute shrinkage and selection operator regularization (LASSO), support vector machine with a linear or radial basis function (rbf) kernel function, and XGBoost algorithms. LASSO is a linear regressor with regularization and auto-descriptors-selected functions, which forces certain coefficients to be zero. The SVM constructs an optimal hyperplane as a decision surface for separating and training the observations. The XGBoost is a decision-tree-based ensemble algorithm with a gradient boosting functionality. XGBoost fits regression ensembles by a gradient descent algorithm that minimizes the loss. The trees of XGBoost are built sequentially: each tree learns from the prior learner and updates the residual errors. The algorithms were run in Python using the XGBoost python package for XGBoost and the Scikit-learn packages for SVM and LASSO.

**Figure 2.**
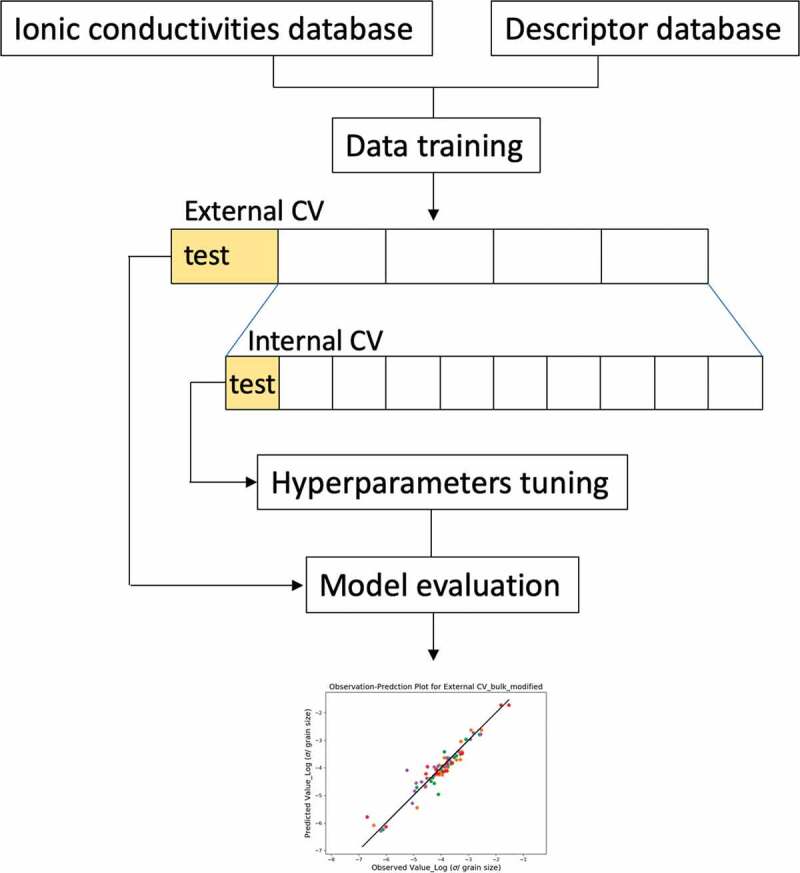
Schematic workflow of machine learning model. The model is trained by two databases composed of ionic conductivities and descriptors via nested cross-validation for hyperparameter tuning and model evaluation

The model construction is illustrated in Figure 2. The entire database consisted of the ionic-conductivity database and the descriptors database, and was trained by nested cross-validation (CV) by different algorithms. The target variable of the model is the logarithm of the bulk ionic conductivity in the unit of S/cm or the grain boundary ionic conductance in the unit of S/cm^2^. Also, the descriptors were standardized using the mean and the standard deviation among the training data except for XGBoost. The data were randomly separated into *k* folds for internal (*k* = 10) and external (*k* =5) cross-validation: one *k*-fold was selected as the test fold and the remaining *k*-1 folds were the training folds. The seeds of the *k*-fold separation were identical for a fair performance comparison of the algorithms. The test–training separation was repeated *k* times, maintaining orthogonality of the remaining test folds and no duplications. During each iteration, the embedded internal 10-fold CV generated appropriate hyperparameters for optimizing the model. Meanwhile, the five test folds of the external CV were used in the MSE and *R*^2^ evaluations (Equations (12) and (13), respectively).

## 3. Results and discussion

### Bulk conductivities ($\sigma {\;}_{b}$) and grain boundary conductance ($h_{gb}$) of single grains

3.1.

The bulk and grain boundary conductivities are usually derived from the Nyquist plot obtained by impedance spectroscopy of polycrystalline materials. The two arcs in the Nyquist plot reveal the different characteristic frequencies of the bulk (high frequency) and grain boundaries (low frequency) [46]. Most experimental ionic conductivities are often resolved by using the measured resistance with the size parameters of bulk and not the grain boundary (as shown in Equations (8) and (9)). It is said that the reported grain boundary conductivities have nothing to do with the real grain boundaries. The reported ionic conductivities include the contributions of all grains or grain boundaries thus having the numbers of grain or grain boundary dependence. Additionally, the thickness of grain boundary is usually unknown, making it difficult to determine the size and the number of grain boundaries. In order to straightforwardly correlate the ionic conductivities with the structural and experimental descriptors, the single bulk and single grain boundary conductivities should be determined.

**Figure 3.**
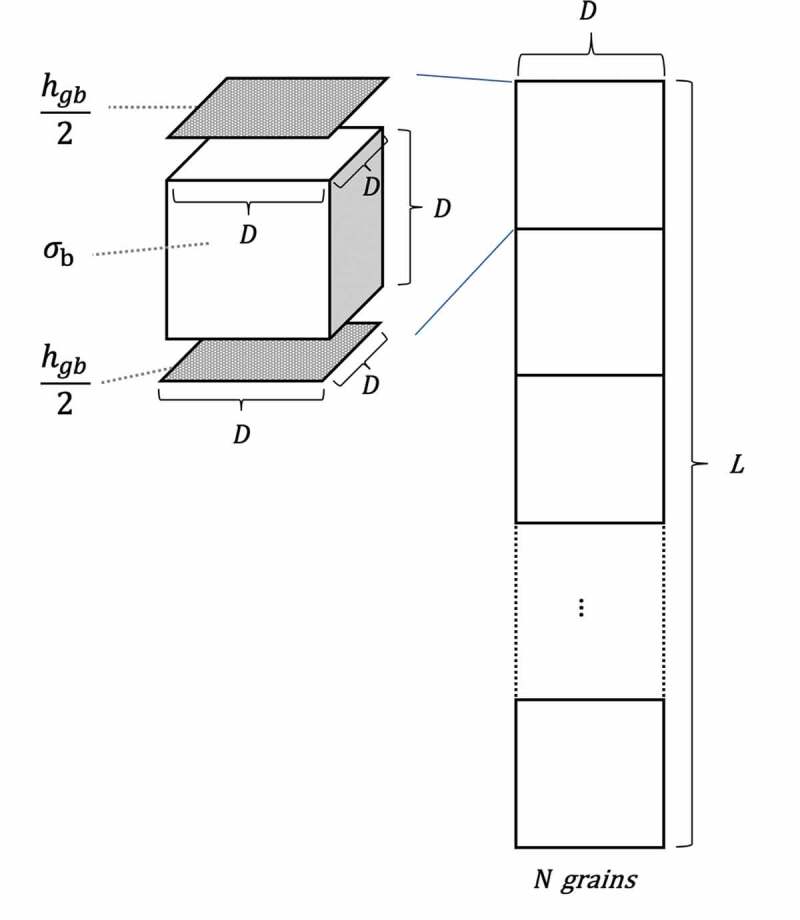
Schematic representations of a model for calculating the bulk conductivities (\sigma _b^) and grain boundary conductance (h_{{\rm{gb}}}^) of single grains. As the grain boundary thickness is much smaller than the grain size (D), it is assumed zero in the model. Thus, the sample length (L) is expressed as L=ND, where *N* is the number of grains along the direction of the heat flow. Each cubic grain has bulk conductivity of \sigma _{\rm{b}}^ (S/cm) and grain boundary conductance of h_{{\rm{gb}}}^/2 (S/cm^2^) of both top and bottom interfaces

Therefore, we propose a one-dimensional model with *zero* grain-boundary thickness for calculating the bulk conductivities (\sigma _{\rm{b}}^) and grain boundary *conductance* (h_{{\rm{gb}}}^) of single grains (see Figure 3) as the grain boundary thickness is much smaller than the grain size. The parallel grain-boundary resistance is neglected, which is appropriate when the grain size is much larger than the grain boundary thickness (as assumed in brick-layer model) [46–48]. In this model, we assume that the sample is composed of cubic grains with side length of *D* and these grains are directly aligned. The sample length *L* is expressed as Equation (3), where *N* is the number of grains along the direction of the electrical current. The total bulk conductivity and total grain boundary conductivity through the whole sample are represented as $\sigma_{b}^{tot}$and $\sigma_{gb}^{tot}$, respectively, and those values are assumed to be obtained through the Nyquist plot. Single cubic grain is illustrated in the left side of Figure 3 and has bulk conductivity of \sigma _{\rm{b}}^ (S/cm) and grain boundary conductance of h_{{\rm{gb}}}^/2 (S/cm^2^) of both top and bottom interfaces. The \sigma _{\rm{b}}^ and h_{{\rm{gb}}}^ are the desired values for further analysis.

(3) L=ND

The total bulk resistance ($R_{b}^{tot})$are N bulk values of each cubic grain ($R_{b})$, and, in the same way as bulk, the total grain boundary resistance ($R_{gb}^{tot})$are N grain boundary values of each cubic grain ($R_{gb})$, as shown in Equations (4) and (5), respectively.

(4) $$R_{b}^{tot}=NR_{b}$$

(5) $$R_{gb}^{tot}=NR_{gb}$$

The bulk and grain boundary resistance of *each cubic grain* ($R_{b},R_{gb}$) can be written in Equations (6) and (7), whereas the total bulk and grain boundary resistance ($R_{b}^{tot},R_{gb}^{tot}$) can be calculated from $\sigma_{b}^{tot}$ and $\sigma_{gb}^{tot}$ as shown in Equations (8) and (9), respectively.

$$\;\;\;\;\;\;\;\;\left\{\begin{array}{c} R_{b}=\frac{1}{\sigma_{b}D}\;\;\;\;\;\;\;\;\;\;\;\;\;\;\;\;\;\;\;\;\;\;\;\;\;\;\;\;\;\;\;\;\;\;\;\;\;\;\;\;\;\;\;\;\;\;\;\;\;\;\;\;\;\;\;\;\;\;\;\;\;\;\;\;\;\;\;\left(6\right)\\ R_{gb}=\frac{1}{h_{gb}D^{2}}\;\;\;\;\;\;\;\;\;\;\;\;\;\;\;\;\;\;\;\;\;\;\;\;\;\;\;\;\;\;\;\;\;\;\;\;\;\;\;\;\;\;\;\;\;\;\;\;\;\;\;\;\;\;\;\;\;\;\;\;\;\;\left(7\right) \end{array}\right.$$

$$\;\;\;\;\;\;\;\;\left\{\begin{array}{c} R_{b}^{tot}=\frac{L}{\sigma_{b}^{tot}D^{2}}\;\;\;\;\;\;\;\;\;\;\;\;\;\;\;\;\;\;\;\;\;\;\;\;\;\;\;\;\;\;\;\;\;\;\;\;\;\;\;\;\;\;\;\;\;\;\;\;\;\;\;\;\;\;\;\;\;\;\;\;\;\left(8\right)\\ R_{gb}^{tot}=\frac{L}{\sigma_{gb}^{tot}D^{2}}\;\;\;\;\;\;\;\;\;\;\;\;\;\;\;\;\;\;\;\;\;\;\;\;\;\;\;\;\;\;\;\;\;\;\;\;\;\;\;\;\;\;\;\;\;\;\;\;\;\;\;\;\;\;\;\;\;\;\;\;\;\left(9\right) \end{array}\right.$$

Since the grain boundary thickness is much smaller than D, it is assumed zero in the model. Then L is equal to the length of N grains (Equation (3)) and the bulk conductivity of each cubic grain (\sigma _b^) equals the total bulk conductivity ($\sigma_{b}^{tot}$) in Equation (10), which is an intrinsic property of the material.

By inserting Equations (3), (6), and (8) into Equation (4), we get:

(10) $$\sigma_{b}=\sigma_{b}^{tot}$$

and inserting Equations (3), (7), and (9) into Equation (5) gives:

(11) $$h_{gb}^{\;}=\frac{\sigma_{gb}^{tot}}{D}$$

Hence, the desired grain boundary conductance of each cubic grain (h_{{\rm{gb}}}^) can be determined by the inverse grain size (D) obtained from the total grain boundary conductivity ($\sigma_{gb}^{tot})$ as shown in Equation (11). The desired \sigma _b^ and h_{{\rm{gb}}}^ were resolved from the reported ionic conductivities ($\sigma_{b}^{tot}$, $\sigma_{gb}^{tot}$) by Equations (10) and (11) for further model training and analysis.

### 3.2 Data distribution

**Figure 4.**
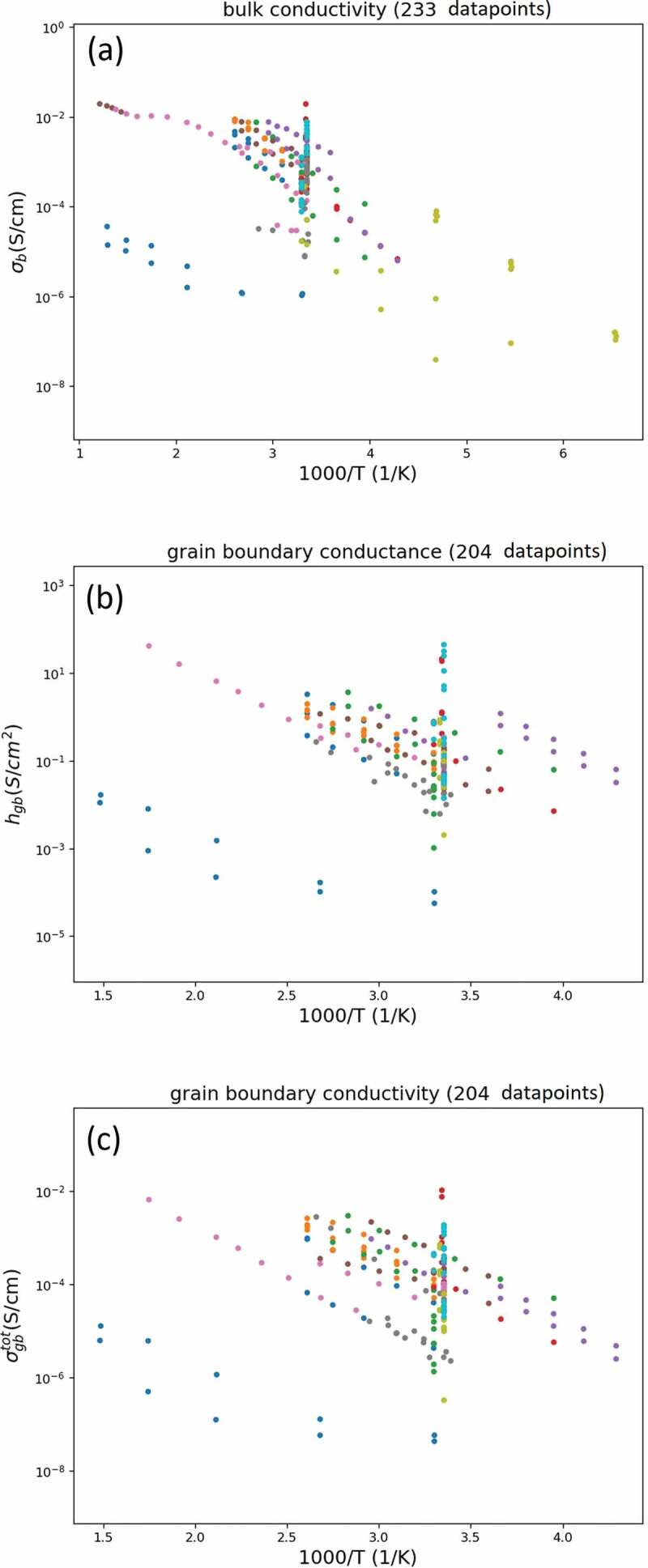
The plots of bulk and grain boundary conductivities against temperature. The (a) desired bulk conductivity of single grains (\sigma _b^), (b) desired grain boundary conductance of single boundaries (h_{gb}^) and (c) observed grain boundary conductivity of total boundaries ($\sigma_{gb}^{tot}$) are colored by various samples

The observed bulk and grain boundary conductivities, which can be considered as $\sigma_{b}^{tot}$ and $\sigma_{gb}^{tot}$, respectively, in Equations (10) and (11), were collected from experimental papers. After selecting the data containing structural and experimental properties such as grain size for deriving the h_{{\rm{gb}}}^, 437 data points (bulk and grain boundaries) composed of 96 different samples at various temperatures are available for further analysis. Further details of the descriptor selection and calculation are provided in the Methods section. The desired \sigma _b^and h_{{\rm{gb}}}^ are plotted as functions of temperature in Figure 4(a,b), respectively. Here, the colors represent the different samples. The desired \sigma _b^ and observed bulk conductivities are identical, whereas the desired h_{{\rm{gb}}}^ is calculated from the observed grain boundary conductivity of total boundaries ($\sigma_{gb}^{tot}$) (see Figure 4(c)). The ionic conductivities in Figure 4 are obviously temperature dependent and follow the Arrhenius law. When investigating the physical effects of the other descriptors on the bulk and grain boundary conductivities, we exclude this strong temperature dependence, and therefore select only one \sigma _b^ and h_{{\rm{gb}}}^ value, measured in the range 24–30°C, of each sample (96 samples) from the dataset for machine learning.

**Figure 5.**
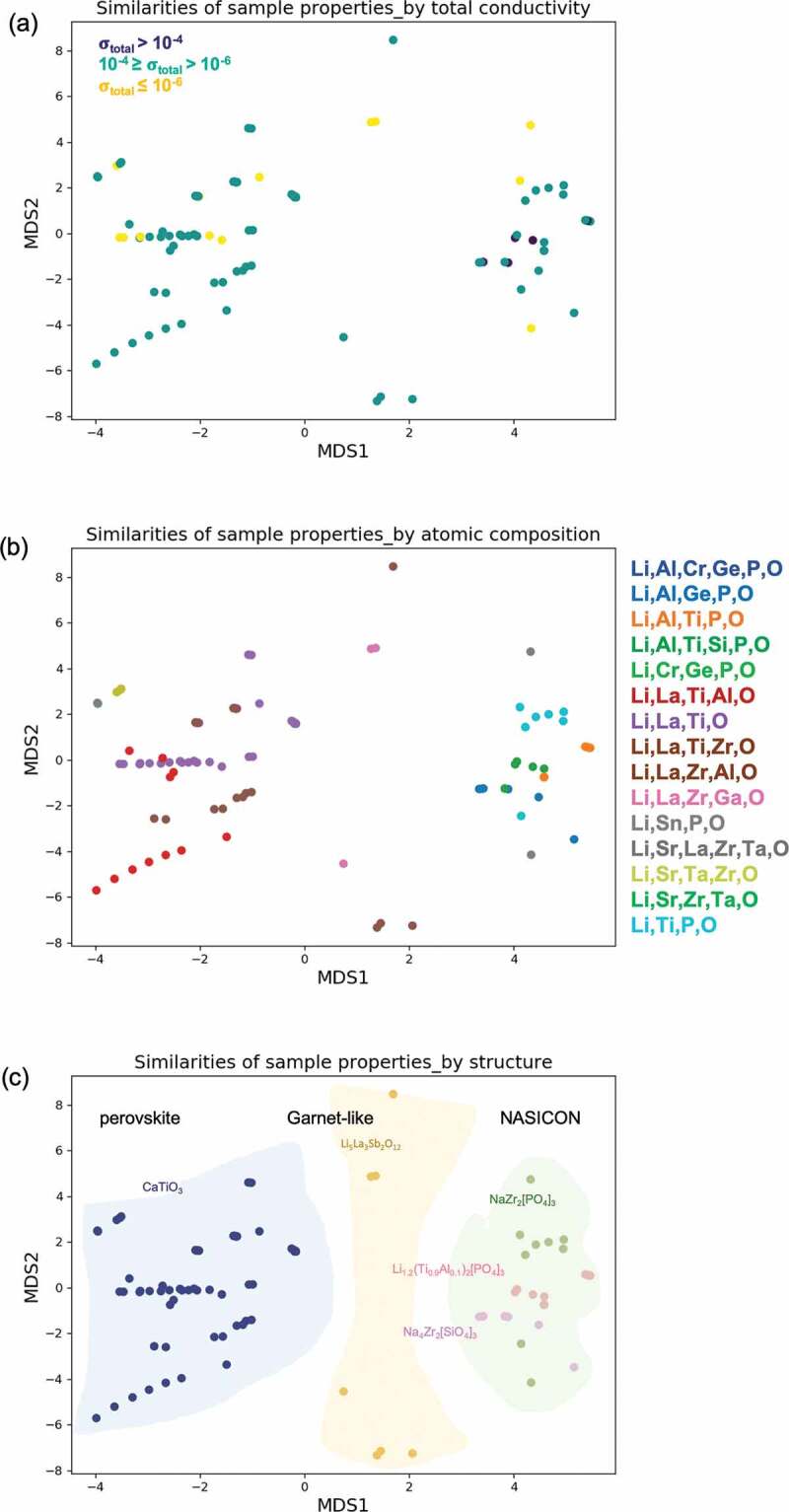
The two-dimensional MDS plots of 96 samples. The similarities are evaluated by Euclidean distance of each sample’s structural, material, chemical and experimental descriptors (17 descriptors are listed in Figure 1). All the 96 samples are colored by (a) total ionic conductivity, (b) atomic composition and (c) structure type. There are 15 atomic compounds in (b) and five structure types which are categorized into three structure classes of perovskite, garnet family and NASICON as blue, yellow and green regions, respectively, in (c)

The similarities of the 96 samples were visualized by two-dimensional metric multidimensional scaling (MDS) [49] (see Figure 5). Using a similarity matrix, MDS plots a series of *n* objects against the coordinates of the same objects in an *m*-dimensional space, where *m* is usually less than three. The similarity matrix is evaluated by the Euclidean distance of all 17 descriptors; a larger distance between two output points implies a higher dissimilarity between the two samples. In Figure 5(a), the samples are colored by their total conductivity (their combined bulk and grain boundary conductivities). Most of the samples had total conductivities of 10^−4^ to 10^−6^ S/cm at near-room temperatures (24–30°C), but five samples had total conductivities above 10^−4^ S/cm (aggregated at the right side of Figure 5(a)). Comparing the MDS plots colored by atomic composition and structure type in Figure 5(b) and (c), respectively, these five highly ionic conductive samples [11,34,35] are of the NASICON structure type, which comprise covalent networks of corners sharing octahedra and PO_4_ tetrahedra providing interstitial sites for the hopping of Li ions. Their atomic compositions included Li/Al/Ti/P/O [34], Li/Al/Ti/Si/P/O [35], Li/Al/Ge/P/O [11] and Li/Cr/Ge/P/O [11]. All of the highly conductive samples were oxides; sulfide ionic conductors such as Li_10_GeP_2_S_12_ (with ionic conductivities up to 10^−2^ S/cm at room temperature, as reported by Kamaya et al.) [50,51] were excluded because those data lacked several items of descriptor information (specifically, the grain size, sintering temperature, or pellet diameter). Figure 5(b) shows 15 different atomic compositions, with common substitute elements of La, Al, Ge, Zr, Ti, and Sr. The structure types of CaTiO_3_, Li_5_La_3_Sb_2_O_12_, NaZr_2_[PO_4_]_3_, Li_1.2_(Ti_0.9_Al_0.1_)_2_[PO_4_]_3_, and Na_4_Zr_2_[SiO_4_]_3_ belonged to three classes: perovskite, the garnet family, and NASICON (labeled in three regions in Figure 5(c)). The materials of the 96 samples and their structure types are listed in the Supplementary Information.

### 3.3. Algorithm selection

The desired \sigma _b^ and h_{{\rm{gb}}}^were predicted using the structural and experimental descriptors in different algorithms. We only used the data measured near room temperature because the ionic conductivity and temperature are related by the Arrhenius equation. Therefore, the temperature during measurement was excluded as a descriptor in this work. It should be noted that the temperature mentioned here is different from the sintering temperature. Here, we trained the model to predict the ionic conductivity by using \sigma _b^ and h_{{\rm{gb}}}^ at near room temperatures on the 96 samples and assessed their predictive performances by the coefficients of determination (*R*^2^) and mean squared errors (*MSE*s). The performance results are listed in Table 1. We suppose that ${\overset{ˉ}{y}}_{i}$ is the mean of the target outcomes $y_{i}$, where *i* = 1, 2, …, *n*. The *MSE* calculates the squared difference between each target ($y_{i}$) and its prediction (${\hat{y}}_{i}$) at point *i*, and averages the results as shown in Equation (12).

(12) $$MSE=\frac{1}{n}\overset{n}{\underset{i=1}{\sum }}{\left(y_{i}-{\hat{y}}_{i}\right)}^{2}$$

**Table 1.** Performances (*R*^2^ and *MSE*) of four algorithms for predicting bulk conductivities and grain boundary conductance (nonlinear XGBoost model gives the best performance). Since the target variables in these models are the logarithm of the original target properties, MSEs are dimensionless values

	Bulk ($\sigma_{b}^{\;}$)	Grain boundary ($h_{gb}^{\;}$)
R^2^		
XGBoost	0.772	0.690
SVM(rbf)	0.697	0.673
Lasso	0.564	−0.009
SVM(linear)	0.531	−0.696
MSE		
XGBoost	0.111	0.302
SVM(rbf)	0.147	0.318
Lasso	0.212	0.981
SVM(linear)	0.229	1.648

The *R*^2^ calculates the ratio of the total sum of the squared errors to the total sum of the squares of the difference from the mean, and subtracts the result from unity (Equation (13)). Simply put, *R*^2^ compares the *MSE* to the variance of the outcome response, and is high when the prediction is good.

(13) $$R^{2}=1-\frac{\sum_{i=1}^{n}{\left(y_{i}-{\hat{y}}_{i}\right)}^{2}}{\sum_{i=1}^{n}{\left(y_{i}-{\overset{ˉ}{y}}_{i}\right)}^{2}}$$

**Figure 6.**
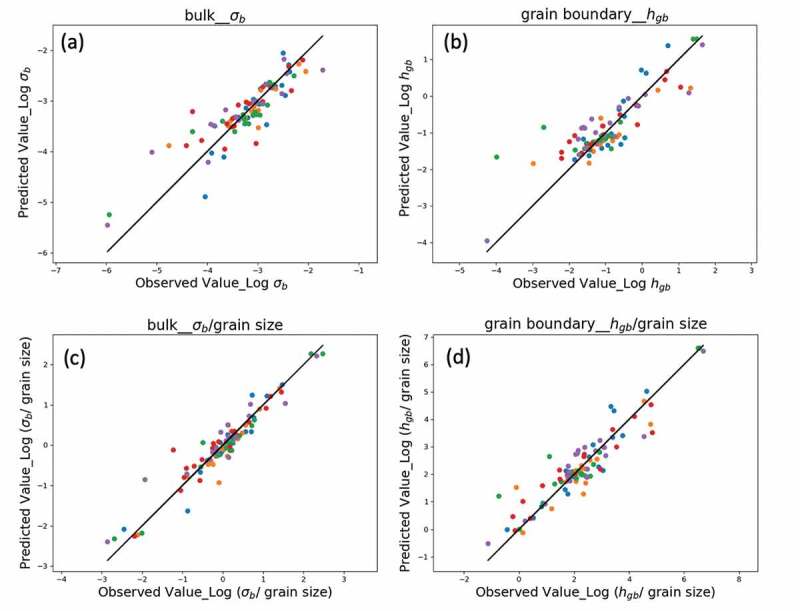
The observation and prediction plots for bulk and grain boundary by XGBoost. The targets of (a) and (b) are the logarithm of bulk conductivities \sigma _b^(S/cm) and single grain boundary conductance h_{gb}^ (S/cm^2^), whereas the newly defined targets of (c) and (d) are logarithm of \sigma _b^/grain size (S/cm^2^) and h_{gb}^/grain size (S/cm^3^), respectively. The five test datasets, which are orthogonal to each other in the external five-fold cross validation and are represented in five different colors, are used for the final evaluation of the model. The details of model construction can be found in Methods section

As seen in Table 1, all *R*^2^ were positive for the bulk materials (up to 0.772), whereas the linear Lasso and support vector machine (SVM) algorithms yielded negative *R*^2^ at the grain boundaries. The negative *R*^2^ shows the poor predictive-performance and basically the model cannot be used. Although the bulk *R*^2^ values of linear Lasso and SVM were comparable to those of the nonlinear XGBoost and SVM (rbf) algorithms, the *MSE*s of the linear algorithms were undesirably high. On this dataset, the nonlinear XGBoost algorithm delivered the best predictive performance (*R*^2^ = 0.772 in bulk, 0.690 at the grain boundaries). The linear algorithms were unsuitable for predicting ionic conductivities, especially at the grain boundaries. The correlations between the observed and XGBoost-predicted conductivities in the bulk and at the grain boundaries are shown in Figure 6(a,b), respectively. The predictions were evaluated by external five-fold cross validation on the test data (in the plots, the test data of each fold validation are represented by different colors). The scattering was smaller in the bulk than at the grain boundary, reflecting the *R^2^* listed in Table 1.

### 3.4. Descriptor analysis of weight and gain importance

The feature importance and predictive performance are required for analyzing the key descriptors of the bulk and grain-boundary conductivities. As the feature importance, which is available for decision-tree-based algorithms, we consider both ‘weight importance’ and ‘gain importance’. The weight importance defines the number of times a feature appear to split the data across all trees, and the gain importance shows the average gain, which contributes to reduce the prediction error, across all splits involving the feature; that is, the weight represents the relative frequency of a feature while the gain implies the relative contribution of a feature in generating the predictive model.

**Figure 7.**
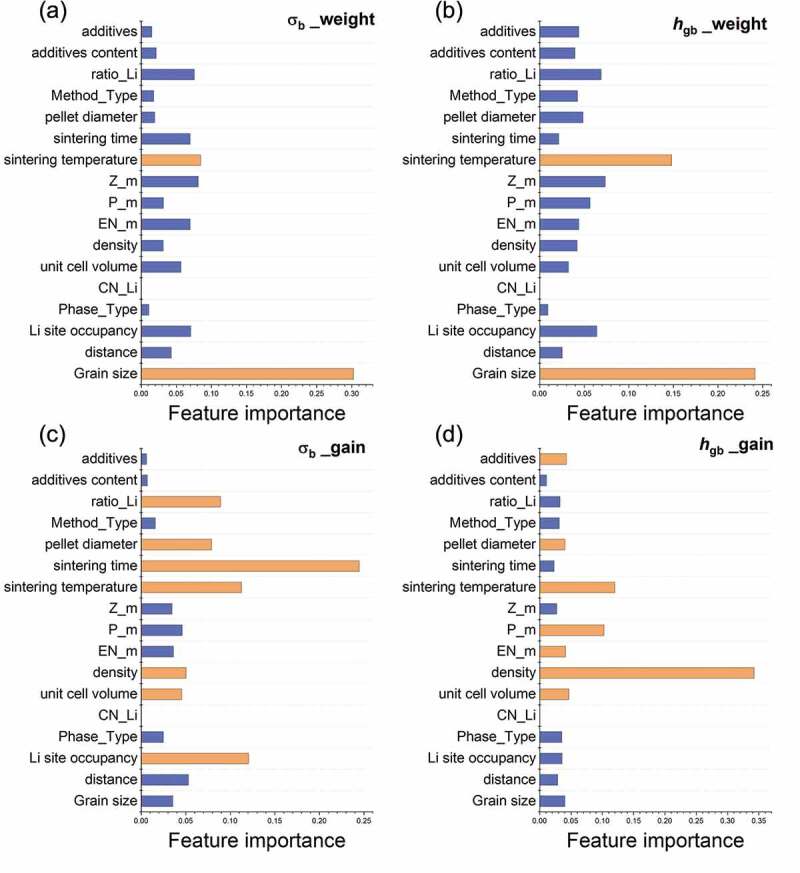
The feature importance for bulk and grain boundary by XGBoost. The importance type of ‘weight’ shows the consistency for (a) bulk and (b) grain boundary while the type of ‘gain’ for (c) bulk and (d) grain boundary reveals the differences of important descriptors. The top-two for weight importance and the top-seven for gain importance represent in orange

**Figure 8.**
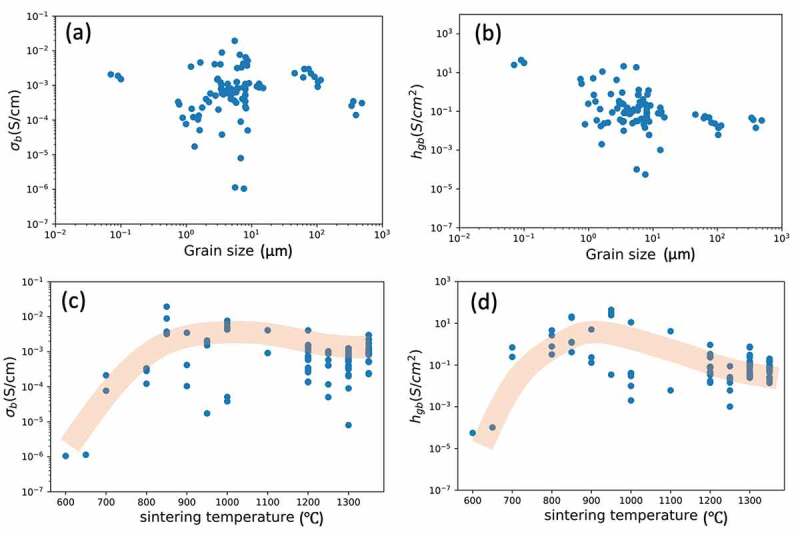
The plots of ionic conductivity of 96 samples at room temperature against the descriptors of grain size and sintering temperature. (a) and (b) show the grain size of the 96 samples with bulk and grain boundary conductivities, respectively. There exhibits a positive correlation between sintering temperature and both (c) bulk and (d) grain boundary conductivities. The orange line is for tendency indicators

Panels (a) and (b) of Figure 7 show the ‘weight importance’ of the features in the bulk and grain boundaries, respectively. The grain size contributed the highest weight, followed by the sintering temperature, in both bulk and grain boundaries. The correlations between the ionic conductivities with the grain size and with the sintering temperature descriptors are shown in Figure 8. The grain size distributed in a large range from 0.07 to 486 μm (Figure 8(a,b)). There is no clear correlation between grain size and ionic conductivities. Regarding to the second-high weight importance of sintering temperature, both the bulk and grain boundary conductivities increased with sintering temperature until the trend converged around 1000°C (Figure 8(c,d) for bulk and grain boundary, respectively).

Nevertheless, we accidentally found a large prediction improvement of the model by using the new targets which calculated by grain size. The new targets were defined by calculating the ionic conductivities using the inverse grain size, namely, \sigma _b^/grain size and h_{gb}^/grain size. Surprisingly, applying the newly defined target of ionic conductivities divided by the grain size, \sigma _b^/grain size and h_{gb}^/grain size, improved the *R*^2^ from 0.772 to 0.897 in the bulk and from 0.690 to 0.818 at the grain boundaries. It should be noted that the improved R^2^ for predicting the new targets mentioned above do not use grain size as a descriptor. The detailed results for predicting new targets could be found in Figure S1 in supplementary. The correlations between the observed and predicted ionic conductivities after applying the newly defined targets are displayed in Figure 6(c) (for bulk) and Figure 6(d) (for grain boundaries). The correlations are clearly improved from those of Figure 6(a,b). The large predictive improvement after applying the newly defined target, obtained by inversely correlating the ionic conductivities with grain size, indicating that the new targets can provide a clearer relationship for the model among targets and other descriptors. It is unclear why the predictive performance was improved after considering the inverse correlation between the ion conductivity and grain size. This phenomenon is worth investigating.

The ‘gain importance’ of each descriptor between the bulk and grain boundary conductivities are demonstrated in Figure 7(c,d), respectively. Among the top seven gain-importance values (labeled in orange in Figure 7(c,d)), the sintering temperature, pellet diameter, density, and unit cell volume are shown for both the bulk and grain boundaries. Regarding to the other descriptors among the top seven, sintering time, Li site occupancy and Li ratio are only shown in bulk, and the additives and chemical properties (polarizability and electronegativity) are only shown at the grain boundaries.

The descriptor with the highest gain importance was sintering time in the bulk, and density at the grain boundaries. The sintering condition reportedly affects the ionic conductivity by changing the microstructural properties such as boundary volume and grain size [4,52,53]. When the dominant phases remain without any phase transitions, defects such as dislocations, stacking faults and pores in the grains are generally reduced during sintering, thus changing the bulk conductivities. The dependence of bulk conductivity on sintering conditions agrees with previous reports [4]. On the other hand, the density gave the highest gain importance at the grain boundaries (Figure 7(d)). It should be noted that our density descriptor defines the density per single crystal, not the density measured in experiments, which can be considered as the density of each grain. The density is an intrinsic property of material that provides the information of the structure class, e.g. the NASICON class has lower density than perovskite and garnet, and both the atoms and their compositions, i.e. the mass per chemical formula unit change with the atomic ratio.

### 3.5. Essential descriptors for the bulk and grain-boundary conductivities

As we discussed in the Introduction section, the material properties, structural properties, chemical characteristics and the experimental conditions are closely interrelated, deconvoluting the correlation for each descriptor with ionic conductivities is not advised. Consequently, the way we interpret the important descriptors from the training models is not to separate each descriptor with given correlation with targets, but to give a hint of which descriptor to tune in priority for bulk or for grain boundary conductivities.

The consistencies (regular) and differences (italic) of the important descriptors between the bulk and grain boundaries are summarized in Table 2. The order of descriptors follows the top-seven gain importance (Figure 7(c,d)), followed by the grain size (no. 8 in Table 2) which is required for determining grain boundary conductance of single grains. Since the gain importance represents the descriptor’s contribution to improve the prediction score, the descriptor with higher gain importance is supposed to have higher decision to control or tune the target even though the correlation with the target is not available (XGBoost is an ensemble non-linear algorithm).

**Table 2.** The important descriptors of the bulk and grain-boundary ionic conductivities. The order is assigned corresponding to each gain importance while the grain size (assigned as no. 8) is necessary for determining grain boundary conductance and provides physical insights such as the thermodynamic condition. Descriptors for both the bulk and grain boundary are represented in a regular font, whereas the individual descriptors for the bulk or for the grain boundary are represented in *italics*, i.e. the sintering time, Li site occupancy, and Li ratio for bulk

	Bulk	Grain boundary
Descriptors	1. *Sintering time*	1. Density
	2. *Li site occupancy*	2. Sintering temperature
	3. Sintering temperature	3. *Polarizability*
	4. *Li ratio*	4. Unit cell volume
	5. Pellet diameter	5. *Electronegativity*
	6. Density	6. *Additives*
	7. Unit cell volume	7. Pellet diameter
	8. Grain size	8. Grain size

Regarding to the results in Table 2, the material descriptors of Li site occupancy and Li ratio dominate the bulk conductivities while the structural descriptors of density and unit cell volume dominate the grain boundary conductivities. The material and structural descriptors used here are the simulated bulk properties from AtomWork-Adv (NIMS, https://atomwork-adv.nims.go.jp/) [45] instead of the measured values. Also, the experimental descriptors of sintering conditions are important for both bulk and grain boundary (within the top-three in Table 2). Furthermore, the additives and the chemical descriptors of polarizability and electronegativity only show for grain boundary. The chemical descriptors are the pseudo-weighted average (see Methods) excluding Li and O ions, suggesting that the grain boundary conductivity is significantly controlled by the additives/dopants which usually compete the diffusive positions with Li ions [54].

From the above, we get the hints for tuning the ionic conductivities: (i) the experimental descriptors of sintering conditions are significant for both bulk and grain boundary, (ii) the material descriptors of Li site occupancy and Li ratio are the prior descriptors, which are material specific, for bulk conductivities, e.g. the samples with the highest bulk conductivity (10^−2^~10^−3^ S/cm, NASICON-type) have Li site occupancy of 0.186–0.375 and Li ratio of ~0.08 in our training dataset. (iii) the density and unit cell volume are the prior structural descriptors while the polarizability and electronegativity are the prior chemical descriptors for tuning grain boundary conductivities, e.g. by various rare-earth doping. It should be remarked again that the tuning way of descriptors is material specific and/or process dependent, e.g. the Li ratio has positive correlation with ionic conductivities for A material but negative for B material.

In regard to the grain size, most importantly, it is required to obtain the meaningful target of grain boundary conductance h_{{\rm{gb}}}^. Additionally, the grain size has the highest weight importance but low gain importance (see Figure 7), which means that the grain size appears many times to split the data but contributes less to the reduction of prediction error. We found that the R^2^ does not change significantly when using the grain size as a descriptor, e.g. R^2^ of bulk conductivities slightly decrease from 0.772 to 0.768 after excluding the grain size as a descriptor. However, the *R*^2^ was remarkedly improved, 0.897 for bulk and 0.818 for grain boundary, by applying the new targets of ionic conductivities divided by the grain size. (See details in Figure S1 in supplementary)

In spite of the low gain importance of grain size, the grain size (i) is necessary for determining grain boundary *conductance* h_{{\rm{gb}}}^ of single grains, which was used as targets instead of the reported grain boundary conductivities ($\sigma_{gb}^{tot}$) for model training (See Equation (11)), and (ii) provides combinational physical insights such as the thermodynamic condition as well as synthesis process. Also, (iii) after applying the newly defined target by grain size, \sigma _b^/grain size and h_{gb}^/grain size, the predictive performance was greatly improved.

The grain size reveals the thermodynamic state of the material under the specific temperature, atomic concentrations, and sintering conditions; for instance, the grain size is usually enlarged after the sintering process. During grain growth, the number of defects such as vacancies inside the grains may decrease, depending on the control of the sintering temperature and atmosphere. After sintering, the microstructural changes in grains with various sizes alter the activation energy of the bulk conductivity; in other words, the least amount of energy required for Li conduction inside the grains depends on the grain size [4]. In a phonon transport study, Xu et al. [41] also reported that the intrinsic thermal conductivity is a function of grain size, and dominates the thermal conductivity of ZnO thin films [55]. Besides the structural changes inside the grains, the region of the space-charge layer (which blocks the ionic conduction) increases rapidly with reducing grain size, thus decreasing the grain boundary conductivities, especially when the grains and the space-charge layer regions are comparable in size [20]. According to the above discussion and results, the grain size effect cannot be excluded when analyzing bulk and grain boundary conductivities.

## 4. Conclusions

We presented a computational (machine learning) approach for identifying the important descriptors for bulk and grain boundary ionic conductivities in lithium solid electrolytes. Both the bulk conductivity and the grain boundary conductance of single grains were derived from 96 samples in three structural classes: perovskite, garnet, and NASICON. The machine learning was performed by the nonlinear XGBoost algorithm while weight importance and gain importance were used for descriptor analysis. Since the material properties, structural properties, chemical characteristics and the experimental conditions are strongly interrelated, we interpret the important descriptors (in Table 2) from the training models by giving a hint of which descriptor to tune in priority for bulk and grain boundary instead of deconvoluting the correlation for each descriptor with ionic conductivities: (i) the experimental descriptors of sintering conditions are significant for both bulk and grain boundary, (ii) the material descriptors of Li site occupancy and Li ratio are the prior descriptors, which are material specific, for bulk conductivities, (iii) the density and unit cell volume are the prior structural descriptors while the polarizability and electronegativity are the prior chemical descriptors for tuning grain boundary conductivities, and (iv) the grain size is necessary for determining grain boundary conductance of single grains and embodies combinational physical insights such as the thermodynamic condition as well as synthesis process. It should be noted that the tuning way of descriptors is material specific and/or process dependent. These findings can clarify ways of improving bulk conductivities and overcoming the limiting factors of grain boundary conductivities.

## Supplementary Material

Supplemental Material

## Data Availability

The materials of the 96 samples and their structure types can be found in the Supplementary Information. Other data that support our findings can be provided by the corresponding authors upon request.
