# Supplementary material for: Essential structural and experimental descriptors for bulk and grain boundary conductivities of Li solid electrolytes
Source: Sci Technol Adv Mater. 2020 Oct 19;21(1):712–25. doi: 10.1080/14686996.2020.1824985 (PMC7594868; doi:10.1080/14686996.2020.1824985)
Supplement: Supplemental Material [file TSTA_A_1824985_SM4336.pdf]

Supplementary

Essential structural and experimental descriptors for bulk and grain boundary conductivities of Li solid electrolytes

Yen-Ju Wu[1,2], Takehiro Tanaka[3], Tomoyuki Komori[3], Mikiya Fujii[*,3], Hiroshi Mizuno[3], Satoshi Itoh[1], Tadanobu Takada[1], Erina Fujita[1], Yibin Xu[*,1]

1) *Center for Materials research by Information Integration (CMI2), Research and Services Division of Materials Data and Integrated System (MaDIS), National Institute for Materials Science (NIMS), 1-1 Namiki, Tsukuba, Ibaraki 305-0044, Japan*
2) *International Center for Young Scientists (ICYS), National Institute for Materials Science (NIMS), 1-2-1 Sengen, Tsukuba, Ibaraki 305-0047, Japan*
3) *Technology Division, Innovation Promotion Sector, Panasonic Corporation, Japan*

Corresponding Authors:

Yibin Xu, Xu.Yibin@nims.go.jp; Mikiya Fujii, fujii.mikiya001@jp.panasonic.com

Contents:

Table S-1 Material list of the 96 samples
Index table 1 Method type
Index table 2 Structure type
Index table 3 Atomic compositions
Figure S-1 Predictive performance of various targets
References

Information Classification: General

Sample	CF	Method_Type	Structure type	Atomic compositions	sintering temperature (°C)	Reference
**1**	Li0.5La0.5Ti1Zr0O3	3	1	7	1300	[1]
**2**	Li0.5La0.5Ti0.98Zr0.02O3	3	1	8	1300	[1]
**3**	Li0.5La0.5Ti0.96Zr0.04O3	3	1	8	1300	[1]
**4**	Li0.5La0.5Ti0.94Zr0.06O3	3	1	8	1300	[1]
**5**	Li0.5La0.5Ti0.92Zr0.08O3	3	1	8	1300	[1]
**6**	Li0.5La0.5Ti0.90Zr0.1O3	3	1	8	1300	[1]
**7**	Li0.5La0.5Ti1Zr0O3	0	1	7	1300	[1]
**8**	Li0.5La0.5Ti0.98Zr0.02O3	0	1	8	1300	[1]
**9**	Li0.5La0.5Ti0.96Zr0.04O3	0	1	8	1300	[1]
**10**	Li0.5La0.5Ti0.94Zr0.06O3	0	1	8	1300	[1]
**11**	Li0.5La0.5Ti0.92Zr0.08O3	0	1	8	1300	[1]
**12**	Li0.5La0.5Ti0.90Zr0.1O3	0	1	8	1300	[1]
**13**	Li0.30Sr0.55Ta0.60Zr0.40O3	0	1	13	1300	[2]
**14**	Li0.35Sr0.475Ta0.70Zr0.30O3	0	1	13	1300	[2]
**15**	Li0.375Sr0.4375Ta0.75Zr0.25O3	0	1	13	1300	[2]
**16**	LiTi2(PO4)3	0	2	15	1000	[3]
**17**	Li1.1Al0.1Ti1.9(PO4)3	0	4	3	1000	[3]
**18**	Li1.2Al0.2Ti1.8(PO4)3	0	4	3	1000	[3]
**19**	Li1.3Al0.3Ti1.7(PO4)3	0	4	3	1000	[3]
**20**	Li1.4Al0.4Ti1.6(PO4)3	0	4	3	1000	[3]
**21**	Li0.375Sr0.438Zr0.25Ta0.75O3	0	1	14	1300	[4]
**22**	Li0.375Sr0.4La0.025Zr0.25Ta0.75O3	0	1	12	1300	[4]
**23**	Li0.375Sr0.3625La0.05Zr0.25Ta0.75O3	0	1	12	1300	[4]
**24**	LiTi2(PO4)3	0	2	15	700	[5]
**25**	LiTi2(PO4)3	0	2	15	800	[5]
**26**	LiTi2(PO4)3	0	2	15	900	[5]
**27**	Li6.25La3Zr2Al0.25O12	0	3	9	1200	[6]
**28**	Li6.35La3Zr2Al0.25O12	0	3	9	1200	[6]
**29**	Li6.55La3Zr2Al0.25O12	0	3	9	1200	[6]
**30**	Li6.76La3Zr2Al0.25O12	0	3	9	1200	[6]
**31**	Li1.7Al0.3Ti1.7Si0.4P2.6O12	2	4	4	900	[7]
**32**	Li1.7Al0.3Ti1.7Si0.4P2.6O12	2	4	4	1000	[7]

Information Classification: General

**33**	Li1.7Al0.3Ti1.7Si0.4P2.6O12	2	4	4	1100	[7]
**34**	Li1.7Al0.3Ti1.7Si0.4P2.6O12	2	4	4	1200	[7]
**35**	Li1.4Al0.4Ti1.6(PO4)3	3	4	3	950	[8]
**36**	Li1.4Al0.4Ti1.6(PO4)3	3	4	3	950	[8]
**37**	Li1.4Al0.4Ti1.6(PO4)3	3	4	3	950	[8]
**38**	LiSn2P3O12	3	2	11	600	[9]
**39**	LiSn2P3O12	3	2	11	650	[9]
**40**	Li0.12La0.75TiO3	3	1	7	1350	[10]
**41**	Li0.16La0.70TiO3	3	1	7	1350	[10]
**42**	Li0.23La0.70TiO3	3	1	7	1350	[10]
**43**	Li0.24La0.65TiO3	3	1	7	1350	[10]
**44**	Li0.33La0.58TiO3	3	1	7	1350	[10]
**45**	Li0.36La0.55TiO3	3	1	7	1350	[10]
**46**	Li0.42La0.52TiO3	3	1	7	1350	[10]
**47**	Li0.09La0.77TiO3	3	1	7	1250	[10]
**48**	Li0.15La0.72TiO3	3	1	7	1250	[10]
**49**	Li0.24La0.65TiO3	3	1	7	1250	[10]
**50**	Li0.31La0.63TiO3	3	1	7	1250	[10]
**51**	Li0.39La0.59TiO3	3	1	7	1250	[10]
**52**	Li0.68La0.49TiO3	3	1	7	1250	[10]
**53**	Li0.49La0.55TiO3	3	1	7	1250	[10]
**54**	Li0.35La0.55TiO3	0	1	7	1350	[11]
**55**	Li0.489La0.592Ti0.956Zr0.044O3.133	0	1	8	1350	[11]
**56**	Li0.383La0.560Ti0.989Zr0.011O3.032	0	1	8	1350	[11]
**57**	Li0.418La0.570Ti0.979Zr0.021O3.064	0	1	8	1350	[11]
**58**	Li0.565La0.615Ti0.932Zr0.068O3.205	0	1	8	1350	[11]
**59**	Li0.35La0.55TiO3	0	1	7	1300	[11]
**60**	Li0.383La0.560Ti0.989Zr0.011O3.032	0	1	8	1300	[11]
**61**	Li0.418La0.570Ti0.979Zr0.021O3.064	0	1	8	1300	[11]
**62**	Li0.489La0.592Ti0.956Zr0.044O3.133	0	1	8	1300	[11]
**63**	Li0.565La0.615Ti0.932Zr0.068O3.205	0	1	8	1300	[11]
**64**	Li1.5Al0.5Ge1.5P3O12	1	5	2	850	[12]
**65**	Li1.5Al0.4Cr0.1Ge1.5P3O12	1	5	1	850	[12]
**66**	Li1.5Al0.25Cr0.25Ge1.5P3O12	1	5	1	850	[12]
**67**	Li1.5Al0.1Cr0.4Ge1.5P3O12	1	5	1	850	[12]

Information Classification: General

**68**	Li1.5Cr0.5Ge1.5P3O12	1	5	5	850	[12]
**69**	LiTi2P3O12	0	2	15	700	[13]
**70**	LiTi2P3O12	0	2	15	800	[13]
**71**	LiTi2P3O12	0	2	15	900	[13]
**72**	Li0.397La0.561Ti0.99Al0.013O3	3	1	6	1350	[14]
**73**	Li0.379La0.562Ti0.99Al0.012O3	3	1	6	1350	[14]
**74**	Li0.366La0.562Ti0.99Al0.011O3	3	1	6	1350	[14]
**75**	Li0.346La0.560Ti0.99Al0.013O3	3	1	6	1350	[14]
**76**	Li1.5Al0.5Ge1.5P3O12	0	5	2	800	[15]
**77**	Li1.5Al0.5Ge1.5P3O12	0	5	2	800	[15]
**78**	Li0.317La0.561Ti0.99Al0.011O3	3	1	6	1350	[16]
**79**	Li0.378La0.562Ti0.99Al0.012O3	3	1	6	1350	[16]
**80**	Li0.342La0.562Ti0.99Al0.013O3	3	1	6	1350	[16]
**81**	Li0.361La0.561Ti0.99Al0.012O3	3	1	6	1350	[16]
**82**	Li0.393La0.56Ti0.99Al0.011O3	3	1	6	1350	[16]
**83**	Li0.41La0.562Ti0.99Al0.013O3	3	1	6	1350	[16]
**84**	Li0.426La0.561Ti0.99Al0.012O3	3	1	6	1350	[16]
**85**	Li0.350La0.557O3	0	1	7	1200	[17]
**86**	Li0.350La0.557O3	0	1	7	1200	[17]
**87**	Li0.303La0.557TiO3	0	1	7	1200	[17]
**88**	Li0.328La0.557TiO3	0	1	7	1200	[17]
**89**	Li0.370La0.557TiO3	0	1	7	1200	[17]
**90**	Li0.303La0.557TiO3	0	1	7	1200	[17]
**91**	Li0.328La0.557TiO3	0	1	7	1200	[17]
**92**	Li0.370La0.557TiO3	0	1	7	1200	[17]
**93**	Li0.35La0.55TiO3	0	1	7	1200	[18]
**94**	Li5.5La3Zr2Ga0.5O12	3	3	10	1100	[19]
**95**	Li5.5La3Zr2Ga0.5O12	3	3	10	1000	[19]
**96**	Li5.5La3Zr2Ga0.5O12	3	3	10	950	[19]

Table S-1 Material list of the 96 samples with each method type, structure type, atomic composition, sintering temperature and reference. The index of method type, structure type, atomic compositions can be found in index table 1, 2, and 3, respectively, as follows. Some samples might have the same chemical formula but are synthetized via different experimental conditions.

Method_Type	index
ball mill, solid phase	0
melt quench	1
liquid phase	2
sol-gel	3

Index table 1

Structure type	index
CaTiO3	1
NaZr2[PO4]3	2
Li5La3Sb2O12	3
Li1.20(Ti0.90Al0.10)2[PO4]3	4
Na4Zr2[SiO4]3	5

Index table 2

Atomic_combination	index
Li,Al,Cr,Ge,P,O	1
Li,Al,Ge,P,O	2
Li,Al,Ti,P,O	3
Li,Al,Ti,Si,P,O	4
Li,Cr,Ge,P,O	5
Li,La,Ti,Al,O	6
Li,La,Ti,O	7
Li,La,Ti,Zr,O	8
Li,La,Zr,Al,O	9
Li,La,Zr,Ga,O	10
Li,Sn,P,O	11
Li,Sr,La,Zr,Ta,O	12
Li,Sr,Ta,Zr,O	13
Li,Sr,Zr,Ta,O	14
Li,Ti,P,O	15

Index table 3

Information Classification: General

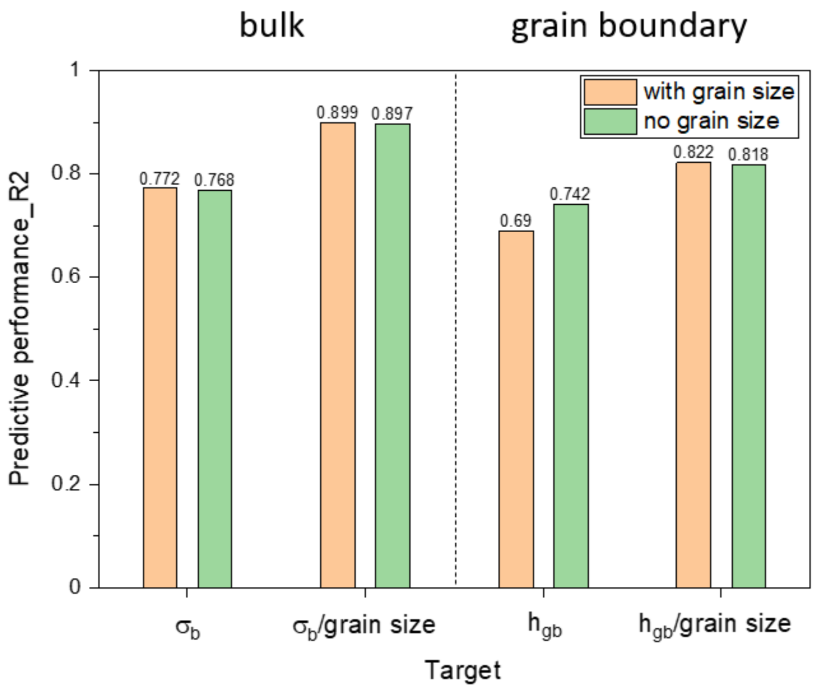

Figure S-1 The predictive performance of R^2 of various targets via XGBoost. The results predicted with and without the grain size as a descriptor are shown in orange and green, respectively. The predictive performance of R^2 does not change significantly when using the grain size as a descriptor, however, the R^2 was improved greatly after applying the new targets of σ_b/grain size and h_{gb}/grain size. σ_{b} is bulk conductivity (S/cm) and h_{gb} is grain boundary conductance (S/cm^2). For all the model training, the logarithms of the original target properties are applied as the target variables.

Information Classification: General

References

1. Ling Me, Zhu X, Jiang Y, et al. Comparative study of solid-state reaction and sol-gel process for synthesis of Zr-doped Li0.5La0.5TiO3 solid electrolytes. Ionics. 2016;22(11):2151-2156.
2. Kimura K, Wagatsuma K, Tojo T, et al. Effect of composition on lithium-ion conductivity for perovskite-type lithium–strontium–tantalum–zirconium-oxide solid electrolytes. Ceramics International. 2016;42(4):5546-5552.
3. Wang S, Ben L, Li H, et al. Identifying Li + ion transport properties of aluminum doped lithium titanium phosphate solid electrolyte at wide temperature range. Solid State Ionics. 2014;268:110-116.
4. Lu J, Li Y. Conductivity and stability of Li3/8Sr7/16-3x/2LaxZr1/4Ta3/4O3 superionic solid electrolytes. Electrochimica Acta. 2018;282:409-415.
5. Kwatek K, Nowiński JL. The lithium-ion-conducting ceramic composite based on LiTi2(PO4)3 with addition of LiF. Ionics. 2018;25(1):41-50.
6. Hao S, Zhang H, Yao W, et al. Solid-state lithium battery chemistries achieving high cycle performance at room temperature by a new garnet-based composite electrolyte. Journal of Power Sources. 2018;393:128-134.
7. Liu M, Li X, Wang X, et al. Facile synthesis and electrochemical properties of high lithium ionic conductivity Li1.7Al0.3Ti1.7Si0.4P2.6O12 ceramic solid electrolyte. Journal of Alloys and Compounds. 2018;756:103-110.
8. Liu X, Fu J, Zhang C. Preparation of NASICON-Type Nanosized Solid Electrolyte Li1.4Al0.4Ti1.6(PO4)3 by Evaporation-Induced Self-Assembly for Lithium-Ion Battery. Nanoscale Res Lett. 2016 Dec;11(1):551.
9. Mustaffa NA, Adnan SBRS, Sulaiman M, et al. Low-temperature sintering effects on NASICON-structured LiSn2P3O12 solid electrolytes prepared via citric acid-assisted sol-gel method. Ionics. 2015 2015/04/01;21(4):955-965.
10. Geng H, Lan J, Mei A, et al. Effect of sintering temperature on microstructure and transport properties of Li3xLa2/3−xTiO3 with different lithium contents. Electrochimica Acta. 2011 2011/03/30/;56(9):3406-3414.
11. Chen K, Huang M, Shen Y, et al. Enhancing ionic conductivity of Li0.35La0.55TiO3 ceramics by introducing Li7La3Zr2O12. Electrochimica Acta. 2012 2012/10/01/;80:133-139.
12. Illbeigi M, Fazlali A, Kazazi M, et al. Effect of simultaneous addition of aluminum and chromium on the lithium ionic conductivity of LiGe2(PO4)3 NASICON-type glass–ceramics. Solid State Ionics. 2016 2016/06/01/;289:180-187.

Information Classification: General

13. Kwatek K, Nowiński JL. Solid lithium ion conducting composites based on LiTi2(PO4)3 and Li2.9B0.9S0.1O3.1 glass. Solid State Ionics. 2018;322:93-99.
14. Le HTT, Ngo DT, Kim Y-J, et al. A perovskite-structured aluminium-substituted lithium lanthanum titanate as a potential artificial solid-electrolyte interface for aqueous rechargeable lithium-metal-based batteries. Electrochimica Acta. 2017 2017/09/10/;248:232-242.
15. Chung H, Kang B. Increase in grain boundary ionic conductivity of Li1.5Al0.5Ge1.5(PO4)3 by adding excess lithium. Solid State Ionics. 2014 2014/10/01/;263:125-130.
16. Le HTT, Kalubarme RS, Ngo DT, et al. Citrate gel synthesis of aluminum-doped lithium lanthanum titanate solid electrolyte for application in organic-type lithium–oxygen batteries. Journal of Power Sources. 2015;274:1188-1199.
17. Hu X, Cheng X, Qin S, et al. Mechanical and electrochemical properties of cubic and tetragonal Li x La 0.557 TiO 3 perovskite oxide electrolytes. Ceramics International. 2018;44(2):1902-1908.
18. Yoon J, Hunter G, Akbar S, et al. Interface reaction and its effect on the performance of a CO2 gas sensor based on Li0.35La0.55TiO3 electrolyte and Li2CO3 sensing electrode. Sensors and Actuators B: Chemical. 2013;182:95-103.
19. Li C, Liu Y, He J, et al. Ga-substituted Li7La3Zr2O12: An investigation based on grain coarsening in garnet-type lithium ion conductors. Journal of Alloys and Compounds. 2017;695:3744-3752.

Information Classification: General
